# Systematic review of surgical treatment for severe elbow osteoarthritis in dogs

**DOI:** 10.1111/vsu.70069

**Published:** 2025-12-12

**Authors:** Alexandria J. Yu, André J. Nault, Wanda J. Gordon‐Evans

**Affiliations:** ^1^ Veterinary Clinical Sciences, College of Veterinary Medicine University of Minnesota Saint Paul Minnesota USA; ^2^ Veterinary Medical Library University of Minnesota Saint Paul Minnesota USA

## Abstract

**Objective:**

To determine the evidence for the most effective surgical treatment for elbow osteoarthritis with the least harm in dogs.

**Study design:**

Systematic review.

**Sample population:**

Peer‐reviewed, English‐language articles describing surgical treatments for elbow osteoarthritis in dogs.

**Methods:**

A literature search was completed using CAB Abstracts, PubMed/MEDLINE, Scopus, and Web of Science on August 19, 2024 for articles describing surgical treatments for elbow osteoarthritis and medial compartment disease in dogs. Inclusion criteria were applied, and the resulting articles were evaluated for level of evidence (Wright scale) and combinability by success rates, and major complications using the number needed to harm (NNH).

**Results:**

Out of the 1231 unique articles, 15 were evaluated based on the inclusion criteria with five prospective studies, eight retrospective studies, and two case series studies. Success rates could not be combined because of variation in outcome reported. Canine unicompartmental elbow had the highest level of evidence for success (91%–98%) with the second best number needed to harm (NNH, 7.6). Sliding humeral osteotomy had the next best evidence with 43%–82% success and 9.5 NNH.

**Conclusion:**

There is low evidence for any of the procedures, and the risk of harm is high.

**Clinical significance:**

Although CUE had the highest level of evidence, there is low evidence overall for efficacy of surgical procedures to treat OA in the elbow. A validated outcome measure with consistent follow‐up intervals to standardized comparisons would allow for better comparison of the outcomes of future studies.

## 1 INTRODUCTION

Elbow osteoarthritis (OA) is common, accounting for the third highest number of joints affected after hips and stifles in a study evaluating 1156 elbows. Of these elbows, 57% had some amount of OA with 16.4% rated as severe.^1^ This can be due to trauma, osteochondritis dissecans (OCD), fragmented coronoid disease (FCP), ununited anconeal process or elbow incongruity. Although each of these diseases have slight variations in initial therapeutic options, the progression of OA continues. The medial compartment tends to be more affected than the lateral because FCP and OCD defects are located on the medial side. This has led to differing types of options for treatment of elbow OA.

Conservative and surgical treatment aims to ameliorate pain. Conservative treatments include weight management, activity modification, use of nonsteroidal anti‐inflammatory drugs (NSAIDs), intra‐articular injections, joint supplements, nonopioid analgesics, regenerative medicine, and laser therapy.^2^ When medical management provides inadequate palliative care, surgical treatments can be performed. There are differing surgical strategies reported in the literature. Surgical strategies that offload weight from the medial compartment by axis‐shifting osteotomies include proximal abducting ulnar osteotomy (PAUL) and sliding humeral osteotomy (SHO).^2^ Another surgical option, called canine unicompartmental elbow (CUE) or partial joint resurfacing, involves the replacement of the articular surface of the medial compartment.^2^ Salvage procedures can be used in these cases or in those where severe OA affects the lateral compartment as well including total elbow arthroplasty (TEA) or arthrodesis may be performed.^2^ This systematic review discusses the different surgical treatments of severe elbow OA to determine the best evidence of short term and long term improvement in clinical signs in dogs with severe OA, including level of improvement and harm.

## 2 MATERIALS AND METHODS

A literature search was conducted on four major science databases, CAB Abstracts, PubMed/MEDLINE, Scopus, and Web of Science using the search term “(dog OR dogs OR canine) AND surg* AND elbow” on August 19, 2024. Broad search terms were used to make sure that reported surgeries were not missed. Article titles were imported into a database and duplicate records were removed. The remaining articles were reviewed and screened based on the following inclusion/exclusion criteria. Records were included when they described in vivo cases with naturally occurring OA with at least 6 weeks of clinical follow up after a surgical procedure. Only articles in English were considered. Articles were excluded if they were evaluating a primary surgical procedure like fracture repair, OCD debridement, or arthroscopy for primary treatment of elbow dysplasia unless a secondary procedure was performed at the same time to directly treat the OA. Proceedings, book chapters, and magazine articles were excluded along with non‐canine species, induced disease, and cadaveric studies. Studies were also eliminated if no clinically relevant functional outcomes were reported (i.e., biomechanical studies or radiographic outcomes only).

Manuscripts that were ultimately included were ranked based on their evidentiary value using the Wright scale^3^ and organized into Table 1 based on surgical treatment performed and measurement of success. The Wright scale ranks the articles based on their study design and places the clinical research study into context for the reader.^3^ There is higher evidentiary value on articles with randomized control study designs and prospective study designs compared to retrospective studies and case series.

**TABLE 1:** Articles included with level of evidence and measurement of success used.

Article	Article number	Surgical treatment	Level of evidence using Wright et al.	Measurement of success used
DeSousa et al.^5^	1	Arthroplasty	III	Surgeon assessment
Conzemius et al.^6^	2		II	Author derived multifactorial
Dinwiddie et al.^7^	3	Arthrodesis	III	Owner satisfaction
McCarthy et al.^8^	4		III	Lameness improvement (LOAD questionnaire, Cook definition^9^)
Silfverberg^10^	5	CUE	IV	DVM evaluation
Bayer et al.^11^	6		III	Author derived multifactorial (LOAD questionnaire, Cook definition,^9^ PE, palpation)
Cook et al.^12^	7		II	Full/acceptable function after surgery
Ballester et al.^13^	8a	PAUL	II	Owner would repeat surgery
Ballester et al.^13^	8b		II	Owner acceptable or better satisfaction
Coghill et al.^14^	9		III	CBPI and NSAID use
Fitzpatrick et al.^15^	10	SHO	III	Lameness
Fitpatrick et al.^16^	11		III	Lameness score <2
McCartney et al.^17^	12		IV	Lameness score ≤3
Wendelburg and Beale^18^	13a		II	PVF‐short term
Wendelburg and Beale^18^	13b		II	PVF‐long term
Quinn and Preston^19^	14		III	Radiographs, second look arthroscopy, clinical findings
Serrani et al.^20^	15	Ulnar osteotomy	II	Lameness score

Abbreviations: CBPI, canine brief pain inventory; CUE, canine unicompartmental elbow; DVM, doctor of veterinary medicine; LOAD, Liverpool osteoarthritis in dogs; NSAID, non‐steroidal anti‐inflammatory drug; PAUL, proximal abducting ulnar osteotomy; PE, physical examination; PVF, peak vertical force; SHO, sliding humeral osteotomy.

Measurement of success was defined a priori as improvement in peak vertical force, owner satisfaction, or surgeon assessment of lameness or clinical improvement. The definition of success was broad because a low number of papers with varying outcome measures was expected. Major complications were defined as adverse events detrimental to long term quality of life, or requiring revision surgery, amputation, arthrodesis, or euthanasia. The percent of success with confidence interval and the number needed to harm (NNH) was calculated for each paper when possible. To calculate NNH, benign neglect was given a value of 0 harm. Data were combined when possible across eligible manuscripts by combining patients treated and calculating success and harm over the entire population treated. Success and harm were reported for short and long term time frames defined as 6 months or less and greater than 6 months, respectively.

## 3 RESULTS

**FIGURE 1:**
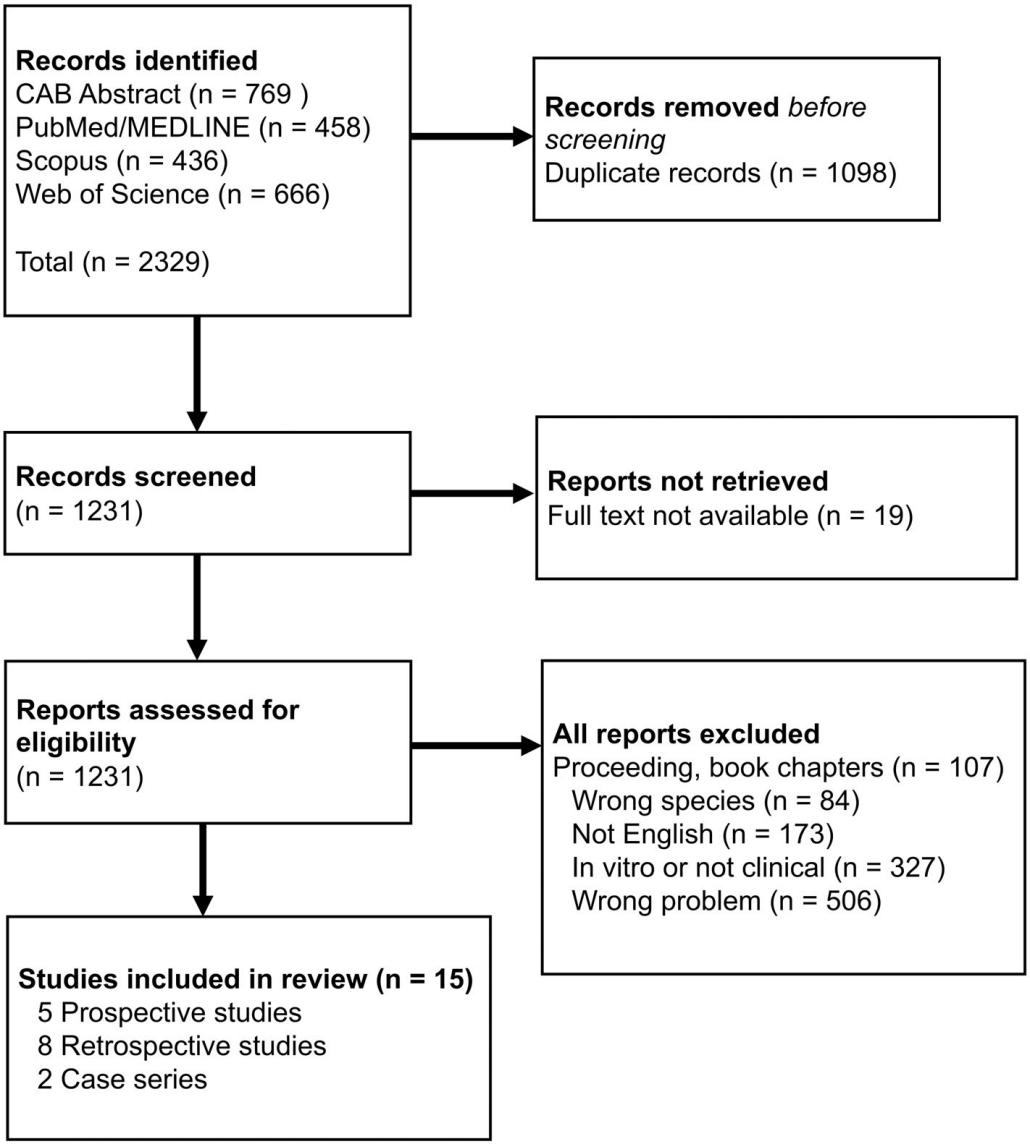
PRISMA flow article identification.^4^

After the initial database search, a total of 2329 records were identified. The search and application of inclusion/exclusion criteria is given in Figure 1 based on PRISMA guidelines.^4^ One article could not be retrieved despite extensive searches and library requests. A secondary screening was applied for eligibility and 36 reports were further excluded after evaluation of the whole manuscript based on the exclusion criteria. Specific reasons for exclusions are listed in Figure 1.

After the identification and screening of the papers, 15 articles were included in this systematic review and ranked on their level of evidence based on the study design (Table 1). None of the included studies provided Level I evidence on the Wright scale. Five papers were prospective studies that provided Level II evidence. Eight articles were retrospective studies that provided Level III evidence and two papers were case series with Level IV evidence. The included papers were organized and grouped according to the surgical treatment performed. Two articles performed arthroplasty, two performed arthrodesis, three papers conducted CUE, two papers performed PAUL, five articles performed SHO, and one paper performed an ulnar osteotomy.

Articles were not combined based on success rate because the criteria was too broad and outcomes disparate and potentially conflicting. However, the percent success is given for each paper. Harm could be quantified across some procedures as most papers defined and reported major complications similarly (Table 2).

**TABLE 2:** Results of applied criteria for success and NNH by surgical method and follow up time.

Procedure	Article	Follow up term	Number of elbows followed	Percent success	Lower CI	Upper CI	Number of complications	Percent Major complications	NNH with 0 as control per surgical treatment group
Arthroplasty	1	Long	33	76%	61.43%	90.57%	14	42.42%	2.9444
	2	Long	20	80%	62.47%	97.53%	4	20%	
Arthrodesis	3	Long	14	78.60%	57.12%	100.08%	12/22	54.54%	1.2667
	4	Long	5	100%	100%	100%	3	60%	
CUE	5	Long	1	100%	100%	100%	0	0%	7.6
	6	Mixed	48	98%	94.04%	101.96%	8	16.67%	
	7	Long	103	91.30%	85.86%	96.74%	12	11.65%	
PAUL	8a	Long	24	74.10%	56.57%	91.63%	4	16.67%	6
	8b	Long	24	88.5%	75.74%	101.26%			
	9	Unknown	30				Not reported		
SHO	11	Short	32	96.88%	90.85%	102.90%	8	25%	3.0714
	12	Short (implied)	5	100%	100%	100%	0		
	13a	Short	6	50%	9.99%	90.01%	6	100%	
SHO	13b	Long	7	43%	6.32%	79.68%			9.5
	14	Long	9				0	0%	
	10	Long	60	81.67%	71.88%	91.46%	0	0%	
Ulnar osteotomy	15	Long	22				≥ 5 (otherwise not explicitly reported)	≥ 22.73%	N/A

Abbreviations: CI, confidence interval; CUE; canine unicompartmental elbow; NNH, number needed to harm; PAUL, proximal abducting ulnar osteotomy; SHO, sliding humeral osteotomy.

## 4 DISCUSSION

Overall, the evidence is weak for use of surgical treatments for elbow OA. CUE had the highest level of evidence for use in dogs with 91%–98% of dogs showing subjective clinical improvement and the second best NNH (7.6). CUE evidence included a prospective observational trial with 103 cases, a retrospective study with 48 cases, and a case report, all of which were assessed subjectively.^9^, ^10^, ^11^ SHO had the next best evidence with 43%–82% success and 9.5 NNH with a small prospective trial with seven long term cases evaluated by peak vertical force and a larger retrospective trial with 60 cases which used lameness as an outcome measure.

Most studies in this systematic review show high levels of success (76%–98%), but also high levels of bias. The authors used a very broad definition of success that was selected to be inclusive of all successful outcomes, but the overall success rate across studies was not calculated because outcome measures were not similar. All studies except one could only be evaluated for success using different methods of subjective assessment. The one paper using objective outcome measures only reported a 43% long term outcome. Thus, these studies may be overstating the success of the procedure. Sometimes even the subjective outcomes were markedly different. For example, Ballester et al. reported two different owner driven outcome measures, the first with an acceptable or better outcome 88.5% but when asked if they would do the surgery again only 74.1% answered affirmatively. This is a large difference in “success” within the same paper.

Furthermore, when using subjective outcomes, it is important to acknowledge that expectations for some surgeries may be different. For example, the dogs that had arthrodesis as a surgical option had previous failed surgeries, which may have made the procedure more difficult or lower in expectation for improvement than if it were a first line surgical option. Lameness is likely also an acceptable outcome because of the mechanical changes to the limb whereas this is not acceptable for a CUE patient. For these procedures, a standardized and validated mobility or quality of life score may be a more appropriate outcome measure for future studies comparing procedures.

Objective outcome measures are ideal in theory as less subjective, but gait analysis is difficult to interpret in dogs with multiple limb abnormalities and adaptations as is common in dogs with elbow OA. Given this constraint, future studies should use a validated instrument to determine outcome with blinded evaluators to limit bias. Strict a priori definitions of success should be defined as well as adverse events to define harm.

The NNH was a much more robust method of combining data for this systematic review. The NNH is interpreted as the number of surgeries performed to harm one additional patient over no surgery. Thus a larger NNH is better. The SHO had the best NNH at 9.5 with CUE at 7.6. However, this is far from ideal as these are major complications. Minor complications were defined too diversely to be used in combination, but would have likely moved the NNH even lower. This means that for every 10 SHO surgeries and every eight CUE surgeries, a patient needed a second surgery or was euthanized. Adverse events are a risk in any surgery, but this highlights the high risk posed by all procedures for elbow OA especially TEA and arthrodesis. The prospective studies were performed early after release of the products in many cases and it is possible that for some surgeons with more experience, the risks are lower.

In this study, NNH was calculated assuming zero harm in a nonsurgical dog. Although it is logical in this particular calculation that dogs that did not receive surgery would not have a major complication, there is likely some harm in leaving dogs painful that have failed conservative management. Randomized, controlled, clinical trials comparing a validated outcome measure for conservative therapy and surgical therapy would be ideal.

This review had some limitations including an English language bias. Papers written in foreign languages were excluded and multilingual searches may have yielded different results. In addition, medical management was not addressed. Treatments for OA have been reviewed by others, and because the surgeries compared here are often considered after clinical signs are refractory to medical treatment, conservative management was not considered for this review.

Overall, this systematic review shows that there is a low level of evidence for all surgical procedures addressing elbow OA, but the best evidence for success while limiting harm is the CUE. Ideally, future randomized, prospective clinical studies that compare the efficacy of surgical treatments to conservative management should be performed with blinded observers if subjective methods are used.

## AUTHOR CONTRIBUTIONS

Yu AJ, BS: Contributed to the study design, conduction of the systematic review, analysis of the included studies, writing of the original draft, and reviewing and editing of the manuscript, and approved the submitted version of the manuscript.

Nault AJ, BSc, MLS: Contributed to the development of the study design and conduction of the systematic review, and reviewing and editing the final manuscript.

Gordon‐Evans WJ, DVM, PhD, DACVS (Small Animal), DACVSMR: Mentor to first author. Outlined the systematic review goals, provided oversight of the project, contributed to the study design, conduction of systematic review, and reviewing and editing of the manuscript.

## CONFLICT OF INTEREST STATEMENT

The authors declare no conflict of interest related to this review.

## Data Availability

All data used in this review is presented in the tables provided.
